# Impact of Aromatic Extracts on Chemical Profile and Sensory Perception of Bread

**DOI:** 10.17113/ftb.63.04.25.9191

**Published:** 2025-12-26

**Authors:** Liege A. Pascoalino, Eliana Pereira, Elisabete Ferreira, Júlia C. Kessler, Madalena M. Dias, Vanessa Vieira, Andreia Afonso, Cristina Gallego, Manuel Gómez, Isabel M. Martins, Lillian Barros

**Affiliations:** 1 CIMO, LA SusTEC, Instituto Politécnico de Bragança, Campus de Santa Apolónia, 5300-253 Bragança, Portugal; 2 Pão de Gimonde, M. Ferreira & Filhas, LDA, EN218, n° 3798, 5300-553, Gimonde, Portugal; 3 LSRE-LCM, ALiCE, Faculty of Engineering, University of Porto, Rua Dr. Roberto Frias, 4200-465 Porto, Portugal; 4 Deifil Technology Lda., Rua do Talho 80, 4830-704 Póvoa de Lanhoso, Portugal; 5 Food Technology Area, College of Agricultural Engineering, University of Valladolid, 34071 Palencia, Spain

**Keywords:** functional food, natural aromas, rosemary extract, almond extract, consumer acceptability, sensory evaluation

## Abstract

**Research background:**

Aromas are known to influence human stimulation, mood and, consequently, food choices and decision-making. In recent years, there has been growing interest in incorporating natural plant-derived extracts into bakery products, not only for their sensory attributes but also for their potential functional value. Among these, rosemary and almond are recognized for their characteristic aroma profiles and bioactive compounds. Bread serves as an attractive vehicle for integrating functional ingredients that can enhance both consumer appeal and product differentiation.

**Experimental approach:**

This work investigates the use of extracts obtained from *Rosmarinus officinalis* L. leaves and *Prunus dulcis* (Mill.) D. A. Webb fruits using supercritical CO_2_ extraction (SFE-CO_2_) as functional food ingredients for breadmaking. Three groups of bread samples were prepared: (*i*) bread without any functionalizing element, the control sample, (*ii*) bread containing rosemary extract (40 µL/kg), and (*iii*) bread containing almond extract (10 µL/kg). Consumer perception of the baked products was evaluated through acceptability tests. The nutritional profile was determined using AOAC methodologies, and the chemical profile was assessed by chromatographic analysis.

**Results and conclusions:**

The nutritional composition of the enriched bread showed no substantial differences compared to the control sample, demonstrating that the addition of the extracts did not alter the fundamental macronutrient profile. In terms of chemical composition, fructose, glucose and maltose were detected, and polyunsaturated fatty acids were the most abundant fatty acids. Importantly, consumer evaluations showed that the inclusion of rosemary and almond extracts improved perception of visual appearance, texture and overall acceptability. These findings indicate that incorporating natural extracts can enhance sensory qualities without compromising nutritional integrity.

**Novelty and scientific contribution:**

This study provides evidence that natural extracts obtained through SFE-CO_2_ can be successfully integrated into bread formulations to enhance sensory appeal while maintaining nutritional stability. The novelty lies in the use of rosemary and almond extracts as functionalizing elements in a staple food matrix, demonstrating their potential to contribute to the development of functional bakery products. This approach provides a sustainable method for diversifying bread formulations, aligning with consumer demand for foods that combine tradition, innovation and health.

## INTRODUCTION

Taste and aroma (the flavour) are undoubtedly key attributes that affect the quality of bread and other baked products (*1*). Among different characteristics of bread, the odour profile is one of the most important, and the pleasant aroma of bread is the parameter that most captivates the consumer. Bread flavour is generally influenced by the choice of ingredients, enzymatic reactions during dough fermentation by yeasts and/or lactic acid bacteria, and thermal reactions during baking. Naturally, any changes in raw materials and ingredients also have an impact on the resulting aroma (*2*, *3*). Thus, aroma is a fundamental feature in bakery products, widely recognized for influencing human stimulation, mood and, consequently, consumer choices and decisions (*4*).

The breadmaking process typically involves three main steps: dough mixing (flour, water, yeast and salt), dough fermentation, and baking. During baking, the starch is gelatinized, proteins are denatured and the raw dough is transformed into a brown-coloured, porous and readily digestible product (*2*, *5*). Although the used ingredients, primarily wheat flour, have their aromatic characteristics, it is generally accepted that the volatile compounds present in flour play a minor role in the overall aroma of bread. These ingredients must undergo significant transformations to develop the distinctive flavour of bread. Prerequisites for the formation of the desired bread flavour compounds are the dough fermentation and baking steps (*2*). Throughout the process, aroma compounds are formed through multiple pathways, including enzymatic activity, microbial fermentation by yeasts and/or lactic acid bacteria, and thermal reactions during baking, most notably caramelisation and Maillard reaction (*2*).

Essential oils (EOs) are highly hydrophobic liquids containing volatile aroma compound or compounds derived from plant materials, including seeds, flowers, leaves, stems, bark, fruits or whole plants (*6*). These oils have the “essence of” the source material in terms of fragrance and are also referred to as volatile oils or ethereal oils (*7*). Traditionally, EOs have been widely used in aromatherapy and conventional treatments for their disinfectant and anti-inflammatory properties. EOs have caught the attention of the research community due to their high phenolic content and diverse bioactivities – including antimicrobial, anticarcinogenic and antihyperglycaemic properties – as well as their numerous other benefits and potential for supportive therapy (*8*–*10*). They have also been recommended as natural food additives for preservation purposes. The growing interest in EOs as natural agents extends not only to the control and prevention of chronic disorders but also to the prevention of decomposition or deterioration of food components (*11*–*13*). Although this is not the focus of the present work, the connection between the antimicrobial properties of EOs and the preservation of bakery products has been previously investigated, with promising results (*8*, *14*–*16*).

Previous works have shown the potential of natural aromas, obtained by supercritical fluid extraction with carbon dioxide (SFE-CO_2_) as solvent, as promising enhancers of the organoleptic properties of bakery products, namely, aromas from *Rosmarinus officinalis* L. (*16*) and *Prunus dulcis* (Mill.) D. A. Webb (*17*), when added to bread dough. These studies primarily focused on olfactory perception as a means to characterise the volatile profile of the resulting bread (crumbs and crusts).

The bread industry has recently evolved in response to growing consumer demand for healthier options and more engaging sensory experiences. As a culturally inherent staple, bread serves as an effective vehicle for sensory and emotional marketing strategies that enhance perceived quality, emotional connection and product appeal. Simultaneously, advances in food science have led to the development of nutritionally improved bread—richer in fibre, protein and functional ingredients—aligning with health and wellness trends. The convergence of sensorial marketing and nutritional enhancement allows producers to influence consumer perception and behaviour while addressing both physical and psychological well-being (*18*, *19*). This integrated approach creates added value by meeting health expectations without compromising sensory satisfaction.

Furthermore, the incorporation of natural ingredients, such as essential oils and aroma-enriched extracts, has emerged as an attractive, exotic and viable alternative in the development of food, cosmetic, aromatherapeutic and pharmaceutical products. The growing consumer preference for natural flavouring agents continues to drive their application in both food and wellness products (*20*).

In contrast, the present study aims to assess the impact of these extracts as food ingredients to enhance the sensory perception of bread from an industrial perspective. Prototypes were prepared and baked in an industrial facility to produce a significant number of samples to be subject to acceptability tests. Furthermore, the resulting bread samples were analysed regarding their chemical (free sugars and fatty acids) and nutritional (moisture, protein, ash, crude fats, total dietary fibre, carbohydrates and energy) profiles. The main objective is to emphasize the potential of natural aromas as clean-label additives that can improve bread aroma while maintaining its nutritional profile.

## MATERIALS AND METHODS

### Sample preparation

#### Extraction of natural aromas by supercritical CO_2_

The natural aromas were extracted from *Rosmarinus officinalis* and *Prunus dulcis species* according to a previously described procedure (*21*). The samples of (30.00±0.05) g were subjected to supercritical CO_2_ extraction (SFE-CO_2_) at 8 MPa and 50 °C for 2 h using a pilot-scale extractor. The chemical composition of the collected aromatic extracts was analysed by GC-MS (TQ8040 NX Triple Quadrupole; Shimadzu, Kyoto, Japan) and their volatile profile was characterised before incorporation into the bread samples (data not shown). The resulting extracts were stored at 4 °C until further use.

#### Bread making and incorporation of natural aromas

All bread samples were produced at the industrial bakery facilities of Pão de Gimonde LDA, located in the village of Gimonde, Bragança, Portugal. The basic recipe follows the traditional “Transmontano” wheat bread formulation, which is composed of flour, water, wheat dough and salt.

Fermentation was carried out using a traditional pre-fermented wheat dough obtained from previous batches, typical of artisanal breadmaking practices in the Trás-os-Montes region. No commercial baker’s yeast was used in the formulation. Wheat flour was supplied by Molinos del Duero i Carbajo Hermanos S.A. (Zamora, Spain) with the following specifications provided by the manufacturer: mass fraction of protein 12 g/100 g and ash <0.8 g/100 g and a falling number >300. The ingredients were mixed for 4 min, after which the respective aromatic extracts, obtained using SFE-CO_2_, were incorporated into the dough. The mixtures were then subjected to an additional 8 min of kneading to ensure uniform distribution of the extracts throughout the dough matrix. The final dough temperature reached 24 °C, and the dough blocks were kept at rest for 60 min. This resting period is essential, since it allows better development of aromas and increases the freshness and durability of the bread. Afterwards, the dough was manually divided into portions (*m*≈600 g), shaped and subjected to a 24-hour resting period in a controlled fermentation chamber at 5 °C and 85 % relative humidity. After the fermentation, the dough samples were removed from the chamber and rested at room temperature ((22±2) °C) for 30 min before baking, allowing them to reach ambient temperature and improve oven spring. To finish this process, baking was carried out in an oven at 240 °C.

**Fig. S1:**
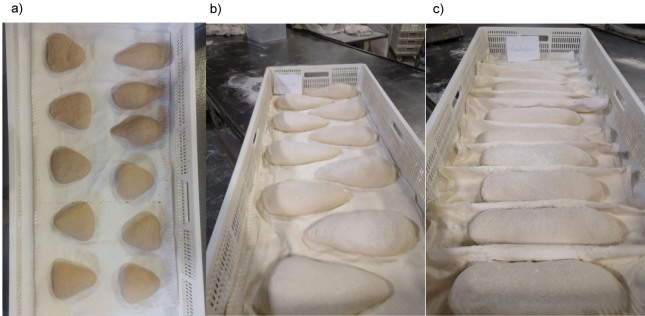
Divided dough samples after 24 h of resting: a) control dough, b) 40 µL/kg rosemary-enriched dough, and c) 10 µL/kg almond-enriched dough

Three sample groups were prepared (Fig. S1): (*i*) control bread without any added extract, (*ii*) bread containing rosemary extract (40 µL/kg), and (*iii*) bread containing almond extract (10 µL/kg). Sensory perception of the baked products was assessed through an acceptability test.

Once in the laboratory, each sample was analysed in triplicate. Prior to chemical and nutritional analyses, the bread samples were lyophilized (LyoQuest Lyophilizer; Telstar, Terrassa, Barcelona, Spain), ground (model A327R1; Moulinex, Barcelona, Spain), homogenized and stored protected from light and moisture.

### Standards and reagents

All chemicals and reagents were acquired from Fisher Scientific (Lisbon, Portugal) and were of analytical grade, except those used for high-performance liquid chromatography (HPLC), which were of HPLC grade.

### Physical analysis

#### Texture

The bread texture profile was determined following a previously described procedure (*22*). The texture analysis was carried out on a Stable MicroSystems (Vienna Court, Godalming, UK) TA.XT Plus texture analyser equipped with a 5 kg load cell and a P/36R aluminium radiused AACC probe. A texture profile analysis (TPA) is a typical test that simulates the chewing of the human mouth by applying two compressions to the sample matrix. The pre-test and post-test speeds were set at 2 and 3 mm/s, respectively, with a target mode of 30 % strain, started at a trigger force of 10 g. The slices of bread used for the texture testing had an approximate thickness of between 15 and 20 mm. The results were processed using a dedicated macro to determine the various dimensions of texture: hardness (N), springiness (%), cohesiveness (%), gumminess (g) and resilience (%). Springiness was determined as the ratio of the distance recovered during the second compression (L_2_) to the distance of the first compression (L_1_) in the texture profile analysis. Values are expressed as percentages, calculated by multiplying the dimensionless ratio by 100, to facilitate interpretation and comparison with literature reports on bread crumb texture. The results were obtained using the Exponent software, v. 6.2 (*23*).

#### Colour

For each sample, colour measurements were taken at three different points on the surface of a bread slice, following a previously reported method (*22*). A portable CR400 colorimeter from Konica Minolta (Chiyoda, Tokyo, Japan) equipped with the D65 illuminant was used. This standard daylight illuminant, defined by the International Commission on Illumination (CIE), is representative of the midday light in Europe. The analysis was conducted using the CIE L*a*b* colour space, where L* represents lightness, a* represents redness (red-green) and b* represents yellowness (yellow-blue). Measurements were performed using a 10° observer angle and 8 mm aperture.

#### pH

The pH of the samples was measured directly using a wireless pH-meter Foodcare HALO® - FC2022 (Hanna Instruments, Woonsocket, RI, USA), which was calibrated before each measurement (*24*).

### Nutritional profile

The bread samples were analysed for key nutritional parameters, including moisture, total protein, ash, crude fats and total dietary fibre content, following the AOAC analytical procedures (*25*). Total available carbohydrates were calculated by difference. The energy value (kJ per 100 g fresh mass) was calculated according to the Regulation (EC) No. 1169/2011 of The European Parliament and the Council (*26*), using the following equation:


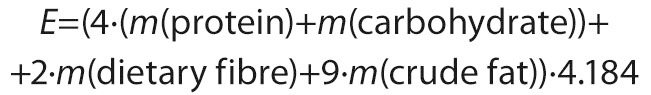
 /1/

### Chemical analysis

For the evaluation of the chemical composition, the mass fractions of soluble sugars and fatty acids were determined according to a previous methods (*27*, *28*). The soluble sugar was determined using a high-performance liquid chromatography coupled to a refraction index detector (HPLC-RI Smartline system 1000; Knauer, Berlin, Germany). Freeze-dried samples were extracted by stirring in a solution of *V*(ethanol):*V*(water)=80 %. Melezitose (25 mg/mL) was used as the internal standard. The sugar mass fraction was expressed in g per 100 g of fresh mass.

The individual fatty acid mass fraction was identified using a gas-liquid chromatography with flame ionization detection (GC-FID) with a capillary column (DANI 1000; DANI Instruments, Contone, Switzerland), following extraction and derivatization to fatty acid methyl esters (FAME). Individual fatty acids were identified by comparing the relative retention times of sample FAME peaks to commercial standards, namely FAME Mix C4-C24 (standard 4788-U; Sigma-Aldrich, Merck, Bellefonte, PA, USA). The results were presented in relative percentages of each fatty acid.

### Sensory analysis

Sensory evaluation of the bread samples was carried out by 86 volunteers (52 females and 34 males), aged between 16 and 65. The bread samples were sliced, each slice coded with four-digit numbers and presented on white plates in random order. Tasters were asked to evaluate appearance, odour, texture, taste and overall acceptability using a 9-point hedonic scale, ranging from 1=dislike very much to 9=like very much. In addition, tasters were also asked to evaluate the intensity of typical bread aromas and any unusual aromas, using a scale ranging from 9=maximum intensity to 1=minimum intensity. The sensory analysis was approved by the Committee of Ethics in the research of the health area of Palencia, Spain (registration no. 2019/026).

### Statistical analysis

For comparison among the samples, one-way analysis of variance (ANOVA) was used, followed by the Tukey’s *post hoc* test. The homogeneity of the variances was also verified. All statistical tests were performed at a 5 % significance level. Data analysis was carried out using the Statistical Package for the Social Sciences (SPSS) v. 24 (*29*). All experiments were carried out in triplicate, and results were expressed as mean value±standard deviation (S.D.), with the number of decimal places adjusted according to the magnitude of the standard deviation.

## RESULTS AND DISCUSSION

### Texture of enriched bread samples

Texture analysis consisted of determining various parameters using the texture profile analysis (TPA) test, which involves a double compression on the sample to mimic the chewing action of the human mouth (*30*). The parameters evaluated were hardness, springiness, cohesiveness, gumminess and resilience, as shown in Table 1.

**Table 1:** Texture parameters of the three bread samples (control and with the addition of 40 µL/kg rosemary and 10 µL/kg almond extract)

Bread sample	Hardness/N	Springiness/%	Cohesiveness/%	Gumminess/g	Resilience/%
Control	(23.5±0.7)^a^	(2.4±0.1)^b^	(0.87±0.03)^a^	(21.1±0.9)^a^	(0.61±0.03)^a^
Rosemary	(11.9±0.5)^b^	(0.98±0.04)^c^	(0.84±0.02)^a^	(10.3±0.4)^b^	(0.51±0.03)^b^
Almond	(11.0±0.3)^c^	(2.8±0.1)^a^	(0.87±0.02)^a^	(9.9±0.4)^b^	(0.58±0.01)^a^

Results are presented as mean value±standard deviation (S.D.), *N*=3. Different letters in the same column indicate statistically significant differences (p<0.05) among samples

Hardness is defined as the force applied by the molar teeth to compress food (*30*) and the results are expressed in Newtons. A previous study (*22*) investigated 5 types of bread and reported a wide range of hardness values, approx. between 5 and 26 N. In the present study, statistically significant differences were observed between (23.5±0.7) N for the control bread to (11.9±0.5) and (11.0±0.3) N for the rosemary and almond-enriched bread, respectively.

Springiness is defined as the rate at which a deformed food returns to its original undeformed shape after the removal of the deforming force, that is, the degree to which the food recovers its height between the first and second bites, expressed as a percentage (*31*). Typically, bread is not considered a highly springy food, and relatively low springiness values are common. The low values observed in this study (ranging from (0.98±0.04) to (2.8±0.1) %) indicate a limited elastic recovery of the crumb structure, which may be related to the dough formulation, fermentation process or addition of aromatic extracts. In this study, the bread enriched with almond aroma exhibited the highest springiness, followed by the control bread, while the rosemary-enriched bread was the least springy, reflecting a slightly greater ability of the crumb to recover its shape after compression. Despite the significant differences among the samples, all showed low springiness values, which are comparable to those reported for various wheat-based breads such as multicereal, Bavaria-style, wholemeal, rye and oat breads in a previous study (*22*, *32*).

Cohesiveness, which is related to the springiness of a food product, is defined as the degree to which a food can be deformed before it breaks (*33*). In this study, cohesiveness was not statistically affected by the addition of aroma-enriched extracts, with values ranging from (0.84±0.02) to (0.87±0.03) %. Similar results have been reported in the literature, with a value of (0.83±0.01) % observed in a study investigating the contribution of the wheat gluten network to the crumb texture of fresh bread (*34*).

Gumminess is defined as the product of hardness and cohesiveness (*31*). The control bread had a significantly higher gumminess value, which can be attributed to its higher hardness value. Finally, resilience refers to the ability of a sample to recover from deformation both in terms of speed and force, and is expressed as a percentage (*31*). Considering the statistical treatment, the variation observed among the analysed bread samples was relatively small, ranging only from (0.51±0.03) % (rosemary) to (0.61±0.03) % (control). Similar results for resilience were previously reported (*22*).

In summary, the texture analysis results suggest that the different aroma-enriched extracts used in the bread formulations did not significantly affect the resilience and cohesiveness parameters. However, they did lead to notable differences in hardness and springiness.

Overall, the texture profile analysis demonstrated that the incorporation of rosemary and almond aromatic extracts significantly reduced the hardness and gumminess of the bread, indicating a softer crumb structure than of the control. The almond-enriched bread showed the highest springiness, reflecting a slightly greater ability to recover its shape after deformation, while rosemary-enriched bread showed the lowest springiness. Cohesiveness and resilience were not significantly affected by the addition of extracts, suggesting that the internal structure and elastic recovery speed of the crumb remained largely unchanged. These findings indicate that aromatic extracts can modify specific textural attributes, improving softness and elasticity without compromising overall crumb integrity.

### Colour of enriched bread samples

Table 2 reports the colour profile of the bread samples, evaluated at three different points on the surface of each bread slice using a portable colorimeter that measured the L*, a* and b* values.

**Table 2:** Colour profile and pH values of the three bread samples (control and with the addition of 40 µL/kg rosemary and 10 µL/kg almond extract)

Bread sample	L*	a*	b*	RGB	pH
Control	(68±1)^a^	(-0.59±0.03)^a^	(15.4±0.3)^a^	173 165 138	(5.44±0.06)^ab^
Rosemary	(68±2)^a^	(-0.71±0.03)^b^	(15.0±0.6)^a^	173 166 139	(5.54±0.03)^a^
Almond	(67±2)^a^	(-0.59±0.03)^a^	(14.2±0.6)^b^	170 163 137	(5.34±0.03)^b^

Results are presented as mean value±standard deviation (S.D.), *N*=3. Different letters in the same column indicate statistically significant differences (p<0.05) among samples. RGB=red, green and blue

The L* parameter represents lightness, where higher values indicate lighter bread. No statistical differences were observed for L*, meaning that the addition of aroma-enriched extracts did not affect the lightness of the bread samples. The a* parameter evaluates the red-green spectrum, with positive values (up to +100 indicating redness) and negative values (down to −100) indicating greenness. All bread samples showed negative values close to 0, with rosemary-enriched bread showing a statistically significantly more pronounced green component. Finally, the b* parameter represents the blue-yellow axis, where positive values correspond to increased yellowness, and negative values to increased blueness. Again, the b* values showed only minor variation, ranging from (15.4±0.3) for the control to (14.2±0.6) for almond-enriched bread.

These results are consistent with findings from other authors. In one study (*34*), cereal bran-enriched bread showed L* values between 69.7 and 51.7, a* values from 7.7 to 14.7 and b* values from 18.5 to 23.6. The higher a* values can be explained due to the substitution of wheat flour for cereal bran. Cereal bran-enriched bread is well-studied in terms of colour, providing a valuable reference point to contextualize the effect of aromatic extracts on bread colour in our study.

Table 2 also presents the corresponding red, green and blue (RGB) colour for each bread crumb sample. Although the differences are subtle, the almond-enriched bread appears slightly lighter in colour. Overall, the statistical analysis showed that, for most parameters, the addition of aroma-enriched extracts did not result in significant colour changes. This suggests that these extracts did not alter the colour of bread, maintaining its acceptability to consumers.

### pH of enriched bread samples

Table 2 shows the pH values measured at three different points on each bread slice. Overall, the bread samples showed slight but statistically significant differences in pH, ranging from (5.54±0.03) for the rosemary-enriched bread to (5.34±0.03) for the almond-enriched bread. The control bread had intermediate values, with no drastic deviation from the other two samples. This indicates that the addition of aroma-enriched extracts did not result in statistically significant alterations in pH. These results are in line with the values of (5.23±0.02) reported in the literature for multigrain bread (*35*).

### Nutritional profile of enriched bread samples

The nutritional profile of the bread samples was also evaluated. The results obtained in the present study showed a very similar nutritional profile among all tested samples (Table 3). Moisture content showed values of approx. 40 %, with no significant differences among the samples. Similarly, no significant differences were found for other parameters, namely ash mass fraction, available carbohydrates and total energy.

**Table 3:** Nutritional values expressed on fresh mass basis of the three bread samples (control and with the addition of 40 µL/kg rosemary and 10 µL/kg almond extract)

Bread sample	*w*/(g/100 g)					*w*(TDF)/%	E/(kJ/100 g)
	Moisture	Protein	Ash	Crude fat	Carbohydrate		
Control	(40±1)^a^	(6.4±0.1)^b^	(1.23±0.03)^a^	(0.16±0.01)^a^	(53±1)^a^	(4.0±0.2)^a^	(961±25)^a^
Rosemary	(40±1)^a^	(6.7±0.1)^a^	(1.20±0.03)^a^	(0.14±0.01)^b^	(52±1)^a^	(3.10±0.03)^b^	(962±13)^a^
Almond	(41±1)^a^	(6.8±0.1)^a^	(1.20±0.03)^a^	(0.13±0.01)^ab^	(51±1)^a^	(5.0±0.2)^b^	(949±21)^a^

Results are presented as mean value±standard deviation (S.D.), *N*=3. Different letters in the same column indicate statistically significant differences (p<0.05) among samples. TDF=total dietary fibre

Protein mass fraction showed slight variations, with an increase in the aroma-enriched bread samples (~6.7 and 6.8 %), compared to the control (6.4 %). The most plausible explanation for the higher protein mass fraction is the preservative effect provided by the natural aroma extracts, which may reduce protein degradation compared to the non-supplemented sample (control). In a previous study (*36*), the use of essential oils was highlighted as a strategy to enhance oxidative stability.

Fat mass fraction was higher in the control sample (~0.16 %), showing a p<0.05 compared to the aroma-enriched bread samples (~0.14 % for rosemary and 0.13 % for almond). Regarding total dietary fibre, the values were ~4.0 % for the control bread, ~3.1 % for the rosemary-enriched bread and 5.0 % for the almond-enriched bread.

Despite the slight statistical variations observed among the analysed bread samples, it can be concluded that the addition of aroma extracts, aimed at enhancing the sensory characteristics, did not significantly compromise their nutritional composition.

### Soluble sugars in enriched bread samples

The soluble sugars were analysed to assess whether the incorporation of aromatic extracts influenced the carbohydrate composition of the bread. While the extracts are not expected to contain notable amounts of sugars, the evaluation allowed the identification of any potential indirect effects resulting from interactions with the dough during fermentation or baking. Regarding the soluble sugars, fructose, glucose and maltose were detected in all bread samples (Table 4) and quantified by comparison with commercial standards and data from the literature. Overall, the total mass fraction of soluble sugars on fresh mass basis was very similar among the samples, with maltose being the most abundant sugar, as expected for cereal-based products (*22*, *31*). Fructose mass fraction ranged from (0.120±0.003) in the control sample to (0.13±0.01) and (0.14±0.01) g/100 g in the almond- and rosemary-enriched bread, respectively. Glucose mass fraction ranged from (0.080±0.003) g/100 g in the almond-enriched bread to (0.10±0.003) and (0.130±0.003) g/100 g in the control and the rosemary-enriched bread, respectively. Maltose, the predominant sugar, showed values of around (2.3±0.1) g/100 g, with no significant differences among samples. Therefore, the addition of aroma-enriched extracts did not significantly affect the content of the major sugar (maltose), and although small variations were observed for fructose and glucose, these were not pronounced enough to alter the overall sugar content. The presence of maltose, fructose and glucose as the main sugars is consistent with the typical composition of regular wheat bread, even when enriched in terms of nutritional profile (*36*).

**Table 4:** Composition of sugars expressed on fresh mass basis in the three bread samples (control and with the addition of 40 µL/kg rosemary and 10 µL/kg almond extract)

Bread	*w*/(g/100 g)		
sample	Fructose	Glucose	Maltose
Control	(0.120+0.003)^b^	(0.100+0.003)^b^	(2.3+0.1)^a^
Rosemary	(0.14+0.01)^a^	(0.130+0.003)^a^	(2.1+0.1)^a^
Almond	(0.130+0.003)^a^	(0.080+0.003)^c^	(2.3+0.1)^a^

Results are presented as mean value±standard deviation (S.D.), *N*=3. Different letters in the same column indicate statistically significant differences (p<0.05) among samples

### Individual fatty acids in enriched bread samples

Table 5 shows the composition of individual fatty acids, expressed as a mass fraction in %. Although saturated fatty acids (SFA) are present in all samples, polyunsaturated fatty acids (PUFA) were the most abundant in all cases; linoleic acid (C18:2n6c) being the predominant PUFA, followed by oleic acid (C18:1n9c). Diets rich in linoleic and oleic acids have been associated with improved blood glucose control, reduced risk of cardiovascular disease and anti-inflammatory effects (*37*). Overall, the almond-enriched bread exhibited the highest PUFA mass fraction and the lowest SFA mass fraction, making it the most nutritionally favourable among the samples. This result is consistent with previous studies (*38*) and is likely due to the naturally high contents of oleic and linoleic acids in almonds. In contrast, the highest mass fractions of saturated fatty acids were found in the rosemary-enriched bread. Among the SFAs, palmitic acid (C16:0) was the most abundant in all samples.

**Table 5:** Individual fatty acid profile, expressed as relative percentage, of the three bread samples (control and with the addition of 40 µL/kg rosemary and 10 µL/kg almond extract)

Fatty acid	*w*(fatty acid)/%		
	Control	Rosemary	Almond
C14:0	(0.55±0.03)^b^	(0.846±0.004)^a^	(0.48±0.02)^c^
C16:0	(20.7±0.5)^ab^	(21±1)^a^	(18±1)^b^
C18:0	(2.4±0.1)^b^	(3.4±0.2)^a^	(2.66±0.01)^b^
C18:1n9c	(30.9±0.5)^a^	(21±1)^b^	(16.7±0.3)^c^
C18:2n6c	(40±1)^c^	(47.7±0.2)^b^	(56.0±0.4)^a^
C18:3n3	(4.6±0.2)^a^	(4.1±0.2)^b^	(3.8±0.1)^b^
C20:0	(0.32±0.01)^b^	(0.51±0.01)^a^	(0.53±0.02)^a^
C20:1	(0.55±0.02)^c^	(0.78±0.02)^a^	(0.652±0.004)^b^
C22:0	(0.38±0.01)^b^	nd	(1.0±0.5)^a^

Results are presented as mean value±standard deviation (S.D.), *N*=3. Different letters in the same row indicate statistically significant differences (p<0.05) among samples. Fatty acids: C14:0=myristic, C16:0=palmitic, C18:0=stearic, C18:1n9c=oleic, C18:2n6c=linoleic, C18:3n3=α-linolenic, C20:0=arachidic, C20:1=eicosenoic, C22:0=behenic. nd=not detected

### Sensory analysis of enriched bread samples

**Fig. 1:**
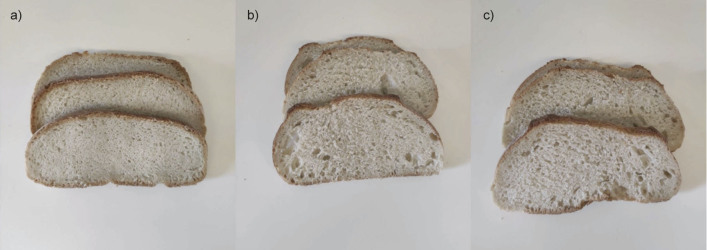
Example of bread samples provided to participants in the sensory tests: a) control, b) 40 µL/kg rosemary-enriched, and c) 10 µL/kg almond-enriched

Fig. 1 shows examples of bread slices provided to the participants during the sensory evaluation. The corresponding results are summarised in Table 6. The panellists did not report statistically significant differences in the intensity of the typical bread aroma among the samples, nor did they detect aftertastes resulting from the addition of the tested aromatic extracts.

**Table 6:** Results of the sensory evaluation of the three bread samples (control and with the addition of 40 µL/kg rosemary and 10 µL/kg almond extract)

	Flavour intensity		Hedonic evaluation				
Bread sample	Bread aroma	Unfamiliar aroma	Visual appearance	Odour	Texture	Taste	Overall acceptability
Control	(6.7±1.8)^a^	(2.3±2.0)^a^	(6.8±1.5)^a^	(6.6±1.7)^a^	(6.0±1.7)^a^	(6.5±1.7)^a^	(6.6±1.4)^a^
Rosemary	(7.0±1.6)^a^	(2.5±1.9)^a^	(7.5±1.3)^b^	(7.1±1.5)^a^	(6.9±1.4)^b^	(6.7±1.5)^a^	(7.1±1.2)^b^
Almond	(6.6±1.8)^a^	(2.5±2.0)^a^	(7.5±1.3)^b^	(6.8±1.5)^a^	(7.1±1.4)^b^	(6.9±1.5)^a^	(7.1±1.1)^b^

Results are presented as mean value±standard deviation (S.D.), *N*=3. Different letters in the same column indicate statistically significant differences (p<0.05) among samples

Although not statistically significant, rosemary-enriched bread samples were perceived by some participants as having a more intense bread-like odour and flavour; a subgroup of testers clearly noted and appreciated these attributes in the rosemary-enriched bread, which is in line with previous findings (*16*).

In terms of overall acceptability, the aroma-enriched bread samples were rated more favourably than the control. However, this improvement was not attributed to aroma or flavour, but rather to differences in visual appearance and texture. Panellists described the enriched bread samples as less compact and somewhat airier, a perception supported by visual observation (Fig. 1) and the results of texture analysis shown in Table 1. These observations suggest that the incorporation of rosemary and almond extracts may have influenced fermentation, particularly gas production and retention. This effect warrants further in-depth investigation.

## CONCLUSIONS

The overall results showed that the addition of natural aroma extracts to bakery products leads to bread with good technological properties and highly appreciated sensory attributes, particularly in terms of taste and aroma. Importantly, these additions did not cause significant alterations in the nutritional and chemical profiles traditionally associated with this type of product. Physical parameters, including texture and colour, were also largely unaffected by the inclusion of aroma-enriched extracts in the dough, suggesting that the fundamental characteristics of the bread were preserved.

Given the increasing expectations of consumers, the bakery industry must continually modernise and innovate. In this context, bread enriched with natural aromas presents strong potential for application. It retains the essential qualities of traditional bread and offers enhanced sensory appeal, particularly of aroma, making it a promising to meet evolving market demands. Moreover, as bread remains a culturally and emotionally significant staple, combining sensory enhancements with nutritional integrity aligns with broader health, wellness and emotional marketing trends. This approach allows manufacturers to respond to consumer desires for well-being and pleasurable eating experiences, reinforcing the role of bread in a modern, health-conscious diet.
